# Ruptured secondary abdominal pregnancy after primary laparoscopic treatment for tubal pregnancy: A case report

**DOI:** 10.1097/MD.0000000000009254

**Published:** 2017-12-15

**Authors:** Duoduo Zhang, Anxia Chen, Yu Gu

**Affiliations:** Department of Obstetrics & Gynecology, Peking Union Medical College Hospital (PUMCH), Beijing, People's Republic of China.

**Keywords:** laparoscopic salpingotomy, persistent ectopic pregnancy, secondary abdominal pregnancy

## Abstract

**Introduction:**

Most secondary abdominal pregnancies happen after spontaneous abortion of tubal pregnancy or ruptured intrauterine pregnancy. However, we presented a case of ruptured secondary abdominal pregnancy after primary laparoscopic treatment of tubal pregnancy.

**Case report:**

The ectopic pregnant lesion in the affected tube was thoroughly removed in the primary laparoscopy, and nothing abnormal was detected in abdomen or pelvis. Beta human chorionic gonadotropin levels dropped significantly after surgery, but the patient came back again for severe abdominal pain with beta human chorionic gonadotropin increasing, and free peritoneal fluid in the pouch of Douglas was detected at ultrasonography. The secondary laparoscopy was done according to the intraperitoneal hemorrhage and unstable vital signs. The secondary pregnancy was found ruptured in the splenic flexure of the colon. Although several cases of secondary abdominal pregnancies were reported in the literature, herein we describe a case secondary to the salpingotomy of the primary tubal pregnancy.

**Conclusion:**

After surgery for ectopic pregnancy, the patient's serum beta human chorionic gonadotropin levels should be closely followed until negative. When persistent ectopic pregnancy was suspected after surgery, physicians should keep in mind a rare possibility of secondary abdominal pregnancy.

## 1 Introduction

Abdominal pregnancy affects 1 in 10,000 gestations with a 6% maternal mortality rate.^[1]^ The trophoblast tissue can be attached to the uterine wall, bowel, mesentery, liver, spleen, bladder, and ligaments, which leads to severe blood loss once invading large vessels.^[2]^ In secondary abdominal pregnancy (SAP), the gestational tissue is expelled spontaneously through the defect in the primary implantation site into the peritoneal cavity, such as tubal abortion or uterine rupture.^[3]^

Laparoscopy is a gold standard for diagnosing and treating ectopic pregnancy nowadays, including conservative salpingotomy and radical salpingectomy.^[4]^ Yet, persistent ectopic pregnancy (PEP) could occur as a complication, as the fallopian tube may result in the persistence of trophoblasts even after the hematoma is removed.^[5]^

By presenting the following case, we would like to emphasize that even a patient has received surgical correction for ectopic pregnancy, serum beta human chorionic gonadotropin (β-hCG) levels should be carefully followed until negative. When PEP is suspected after surgery, doctors should better keep in mind of a rare possibility of SAP occurrence.

## 2 Case report

A healthy 28-year-old nulligravida woman presented to the emergency department complaining of irregular vaginal spotting for 1 week and progressive lower abdominal pain for 1 day. She was trying to conceive during recent months and had her last menstruation 40 days before admission. β-hCG level measured was 3227 IU/L. Transvaginal ultrasound (TVS) showed a normal empty uterus and endometrium measuring 2.7 cm; an enlarged mixed echo nodular, measuring 6.2 cm × 4.3 cm × 3.0 cm, was found in right adnexa; a hypoechoic, measuring 5.9 cm × 3.2 cm, presumably blood, was scanned in the pouch of Douglas (POD). After admission to the gynecologic ward, an explorative laparoscopy was performed as tubal pregnancy was highly suspected.

**Figure 1:**
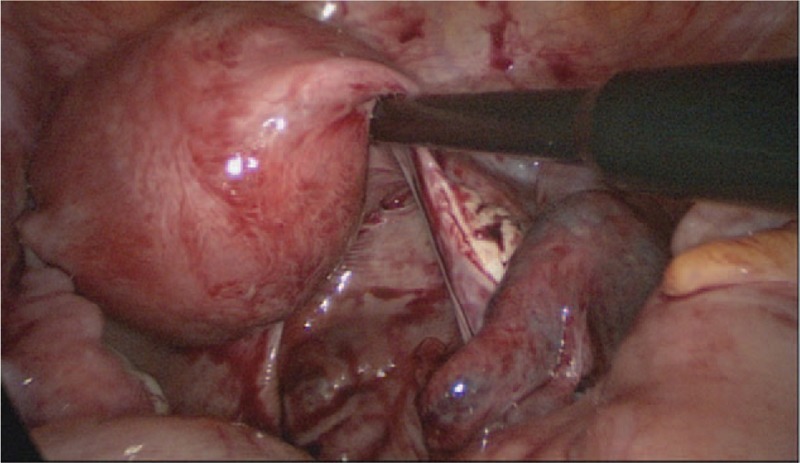
The right tubal pregnancy in the primary laparoscopic surgery.

A purplish mass was found at the ampulla of right fallopian tube during surgery, 3 cm in diameter, and about 200 mL blood in the POD (Fig. 1). A conservative salpingotomy was done. The embryonic tissue and coagula were taken out and the wound was coagulated by monopolar. Both opened tube and the peritoneal cavity were washed thoroughly without any macroscopic tissue or blood left. Histological examination found chorionic villi in the removed tissue. β-hCG decreased to 1734 IU/L in the first day and 340 IU/L in day 3 postoperation. The patient was discharged and asked to follow-up β-hCG level at clinic.

She visited the clinic on day 5 after surgery and the test result showed that β-hCG further decreased to 145 IU/L with nothing abnormal. The patient was asked to follow-up every week till β-hCG was negative; however, she only came back to emergency department after another 10 days when she suffered from a severe “stomach pain.” TVS showed an iso-echoic area in the right adnexa with little blood flow signal; the left adnexa and uterus were clear; a 3.6 cm hypoechoic area was noticed in POD. Blood test showed β-hCG 874 IU/L and hemoglobin 115 g/L. Six hours later, the patient's hemodynamics became unstable with β-hCG 771 IU/L and hemoglobin 99 g/L. Another TVS was ordered and the liquid collected in POD grew to 7.5 cm in depth. So the doctors suspected that a persistent ectopic pregnancy in the right tube was ruptured and the patient received another emergency laparoscopy.

**Figure 2:**
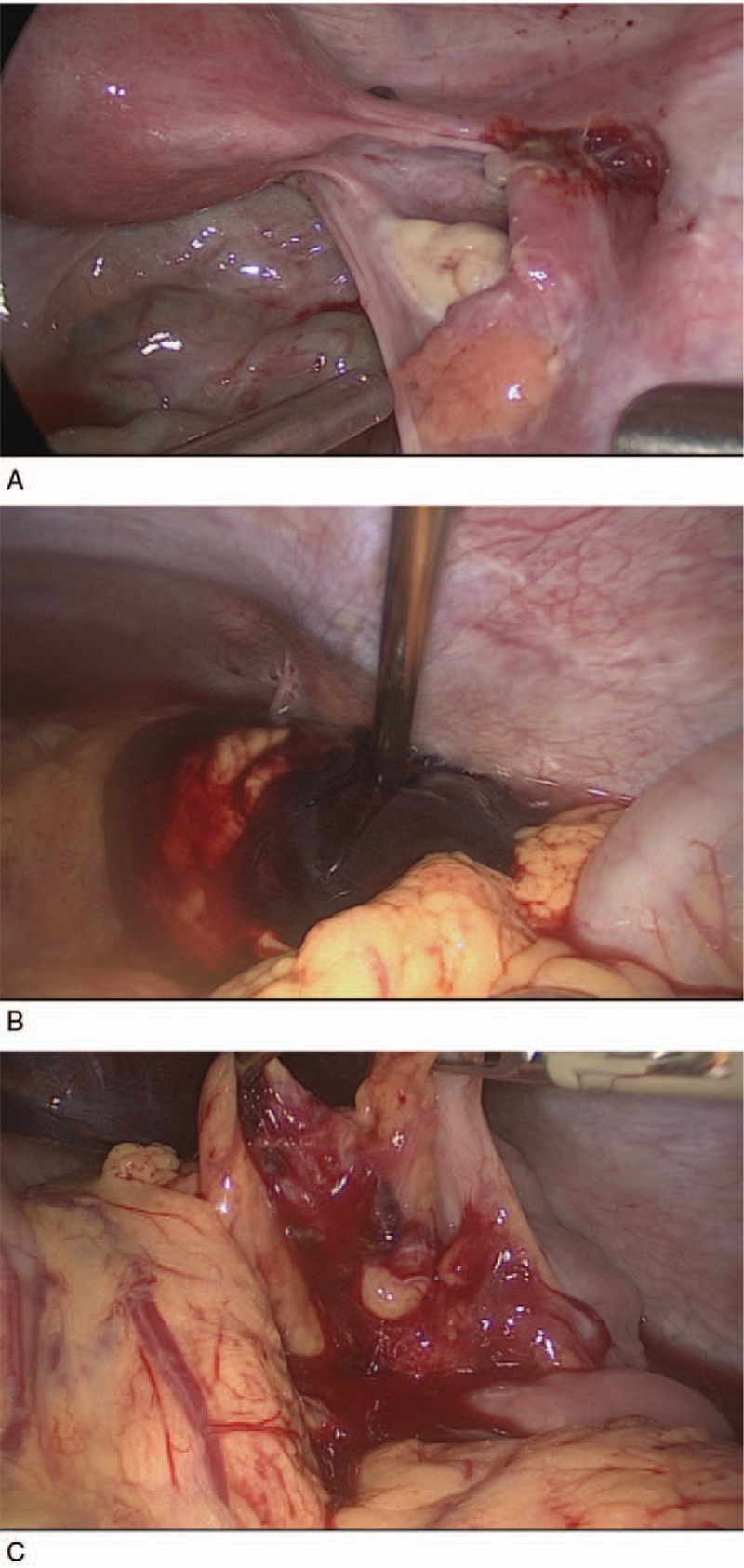
Second laparoscopic surgery. (A) a normal postoperative appearance of right salpingotomy. (B) A bleeding mass attached to the splenic flexure of colon and its mesentery. (C) The wound and ruptured vessel in the mesentery after removal of the mass.

During the surgery, a total of 1000 mL blood and coagula piled in the abdomen cavity; however, all the internal genital organs seemed healthy. Only the right fallopian tube turned out a normal appearance after salpingotomy without any new mass or bleeding wound (Fig. 2A). After a complete wash and careful examination of the abdomen and pelvis, a mass of 5 cm in diameter, attaching the splenic flexure of the colon and its mesentery, was found bleeding actively from its root (Fig. 2B and C). Then the mass containing coagula and some villi-like tissue was removed. Apart from this, a ruptured mesenteric vessel under the mass was ligated. Most of the lesion was located in the mesentery, and only a little bit invaded the seromuscular layer of the colon. During the operation, 500 mL autologous blood salvage, 2 units of condensed red blood cell, and 200 mL serum were transfused.

After surgery, the patient was injected 75 mg methotrexate (MTX) immediately when she came back to the ward. β-hCG continuously declined from 307 IU/L on the postoperative day 1 to 216 IU/L on day 4. However, β-hCG levels remained in a plateau (213 IU/L) on day 5. As a result, another 75 mg MTX was prescribed in case of PEP. On day 7 postoperation, β-hCG dropped to 148 IU/L and the patient was discharged. Pathology reported chorionic villi in the removed tissue. β-hCG kept decreasing and reached negative after another 17 days.

## 3 Discussion

This patient experienced an increasing of β-hCG after laparoscopic removal of tubal pregnancy lesion. No macroscopic lesions were left in surgery; however, they all suffered from ruptured SAP. Postoperative β-hCG levels that were stable or even increased are characteristics of persistent trophoblast activities.^[6]^ The incidence of postoperative PEP was reported as 2.1% to 5.4%.^[5,7]^ Most of these scenarios occurred in situ. Here, we reported a very rare case of postoperative SAP. It was impossible for us to distinguish whether the new lesion was iatrogenic spreading of the embryonic tissue or secondary to the tubal abortion, but we could tell the trophoblastic cells were in high activity. No matter it is a PEP or SAP, follow-up serum β-hCG levels is considered as a unified standard for discovering the lesion as early as possible, even though the patient has received radical removal of the affected salpinx. Postoperative serial monitoring of β-hCG values is especially required after conservative salpingostomy since trophoblastic cells would be left in 5% to 20% women.^[7]^ For salpingectomy, the gestational tissue was leaked out by washing, clamping the tube, or by spontaneous abortion. Our patient ended up with SAP rupture because β-hCG levels were not closely followed until they were negative after her first surgery.

Peritoneal washings are routinely performed in the intraoperative cleaning of left tissue, blood, or coagula, cases of trophoblastic cell or decidua tissue found in peritoneal washing products have been only sporadically described.^[8]^ These cells are well differentiated and in benign condition, but they would change into more invasive architecture in condition of imperfect decidua tissue for nourishing. The washing procedure could not be blamed for the spreading of trophoblastic cells and SAP yet. We still need more evidence on whether it is safe to wash abdomen and pelvis during laparoscopic salpingostomy and salpingectomy.

Most of the reported abdominal pregnancies are secondary to the tubal abortion or rupture of defected or deformed gestational uterus.^[9]^ Ultrasound remains the main method for the diagnosis of extra uterine pregnancy; however, very early pregnancy without a gestational sac or fetus can hardly be detected in the abdominal cavity.^[10]^ This patient complained of a stomach pain, which is quite confusing for a gynecologist. Although her TVS found the internal genital organs were clear, a combination of previous slapingotomy history and an increasing level of β-hCG hinted a possible SAP. As a result, clinicians should pay attention to the exact location of the pain when β-hCG is positive.

Methotrexate is the most common treatment for PEP, and usually only a single dose is required when β-hCG is below 1375 IU/L.^[11]^ The patient received an injection of MTX immediately after surgery, because it was suspected if any high active trophoblast cells were left in the abdomen, but this should be emphasized as just an experimental management. After surgical and medical treatment, β-hCG decreased considerably until postoperative day 5, when it turned out to be a plateau, so a repeated dose was given. The success of MTX treatment does not depend solely on β-hCG levels, and the levels in 1 and 2 doses of MTX patient groups are close to 2000 IU/L with no significant difference, which demonstrates the complexity of PEP.^[5]^

## 4 Conclusions

In conclusion, SAP after primary laparoscopic treatment of tubal pregnancy is very rare. Some imperfections were present in this case, which would have been modified to avoid the severe consequences. We hope the presnt case report increased our awareness towards the following: serum β-hCG levels should be closely and regularly followed after surgical treatment of ectopic pregnancy until it is negative; SAP could be a possible scenario if β-hCG levels decrease unsatisfactorily or even increase after the primary ectopic pregnancy surgery.
